# Gallbladder adenocarcinoma diagnosed retrospectively after initial resection of abdominal wall adenocarcinoma with unidentified primary source: a case report and review of the literature

**DOI:** 10.3389/fonc.2025.1609239

**Published:** 2025-06-06

**Authors:** Kendall Vignaroli, Kevin Perez, Angel Guan, So Un Kim, Sharmila Raju, Carolyn Leach, Judi Anne B. Ramiscal

**Affiliations:** ^1^ Department of Surgical Oncology, Arrowhead Regional Medical Center, Colton, CA, United States; ^2^ Department of Laboratory Medicine, Arrowhead Regional Medical Center, Colton, CA, United States

**Keywords:** gallbladder carcinoma, metastasis, incisional site seeding, abdominal wall mass, delayed diagnosis

## Abstract

Gallbladder carcinoma is the most common malignancy found within the biliary tract and is most often diagnosed incidentally after cholecystectomy is performed. Port-site metastasis discovered after removal of gallbladder carcinoma is not entirely unusual; however, recommendations for the definitive management of these metastasis sites do not yet exist. We report a rare case of incisional site seeding diagnosed prior to the discovery of gallbladder adenocarcinoma in a healthy 59-year-old woman with a history of open cholecystectomy performed in a different country. Ultimately, this patient’s case was staged as stage IVb disease as determined by an extensive multidisciplinary tumor board discussion and was managed with observation, frequent physical exams, and surveillance imaging. Our patient’s survival is 2 years 7 months to date after index cholecystectomy. The management of our unprecedented case of gallbladder carcinoma diagnosed retrospectively after the initial discovery of incisional site metastasis is a difficult one, and the management of unique oncologic scenarios should utilize a multidisciplinary tumor board approach.

## Introduction

Gallbladder carcinoma is a rare cancer of the gastrointestinal system but the most common malignancy within the biliary tract (1). The vast majority of gallbladder cancer cases are discovered incidentally at the time of specimen review after cholecystectomy. In a case review series of 139 patients with gallbladder carcinoma, only six were identified prior to gallbladder removal (2). Consequently, gallbladder cancers are often discovered in advanced stages where resection may no longer be an option, as high as 75% (1). As the majority of cholecystectomies are now performed laparoscopically, there have been cases reported of laparoscopic port-site seeding. A small retrospective review showed two out of 10 patients who presented with obvious tumor growth within the previous laparoscopic tracts at the time of referral for definitive surgical resection (3). An international survey of 607 surgeons reported 409 cases of incidental adenocarcinoma from a total of 117,840 laparoscopic cholecystectomies performed. Of those 409 cases, 70 were reported to have had tumor seeding of the port site during gallbladder removal (4). Based on this survey, the risk of port-site seeding could be extrapolated to be roughly 17%, which is consistent with historical data estimating a risk of 14%–30% (5). However, a systematic review of more recent data from Preferred Reporting Items for Systematic reviews and Meta-Analyses (PRISMA) illustrated a port-site metastasis incidence of 10.3% since 2000 compared to 18.6% from data prior to 2000 (5).

While the occurrence of port-site metastasis is known, the management and subsequent risk-reduction strategies remain controversial. A review of French hospitals over one decade compared patients with gallbladder adenocarcinoma who underwent surgery for curative intent with port-site excisions versus those without and found no survival benefit in patients who received prophylactic port-site excision (6). In a review published in the *Annals of Surgical Oncology*, port-site metastasis was seen exclusively in patients with T2 or T3 disease and was strongly correlated with peritoneal metastasis (7). Furthermore, T2 disease portends a significantly worse prognosis than a T1 tumor, with a sharp drop in 5-year survival from 50% to 28% (8). Given this correlation, the discovery of port-site metastasis is still an important factor in overall treatment prognosis and may give impetus to further investigate peritoneal metastasis. This would be especially important in a case where the primary cancer is not known at the time an abdominal wall cancer is found. We present a unique case of incisional site metastasis found in a patient who underwent open cholecystectomy in a different country in which the diagnosis of gallbladder cancer had been unknown at the time of presentation.

## Case description

**Figure 1:**
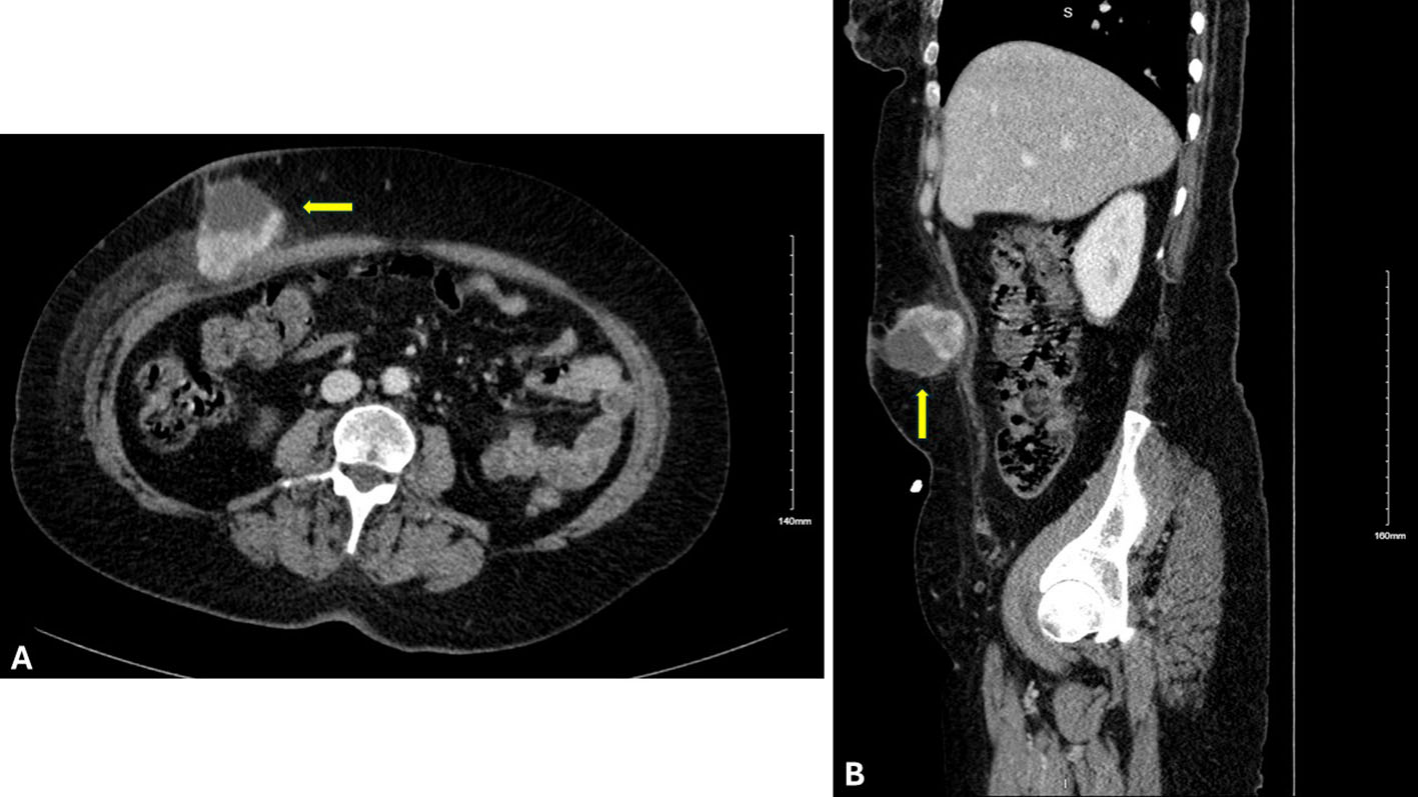
Cross-sectional imaging of the abdomen and pelvis with IV contrast. **(A)** Axial and **(B)** sagittal views displaying a lesion (indicated by the yellow arrow) 8 × 6 × 3.5 cm within the right mid-abdominal ventral wall.

**Figure 2:**
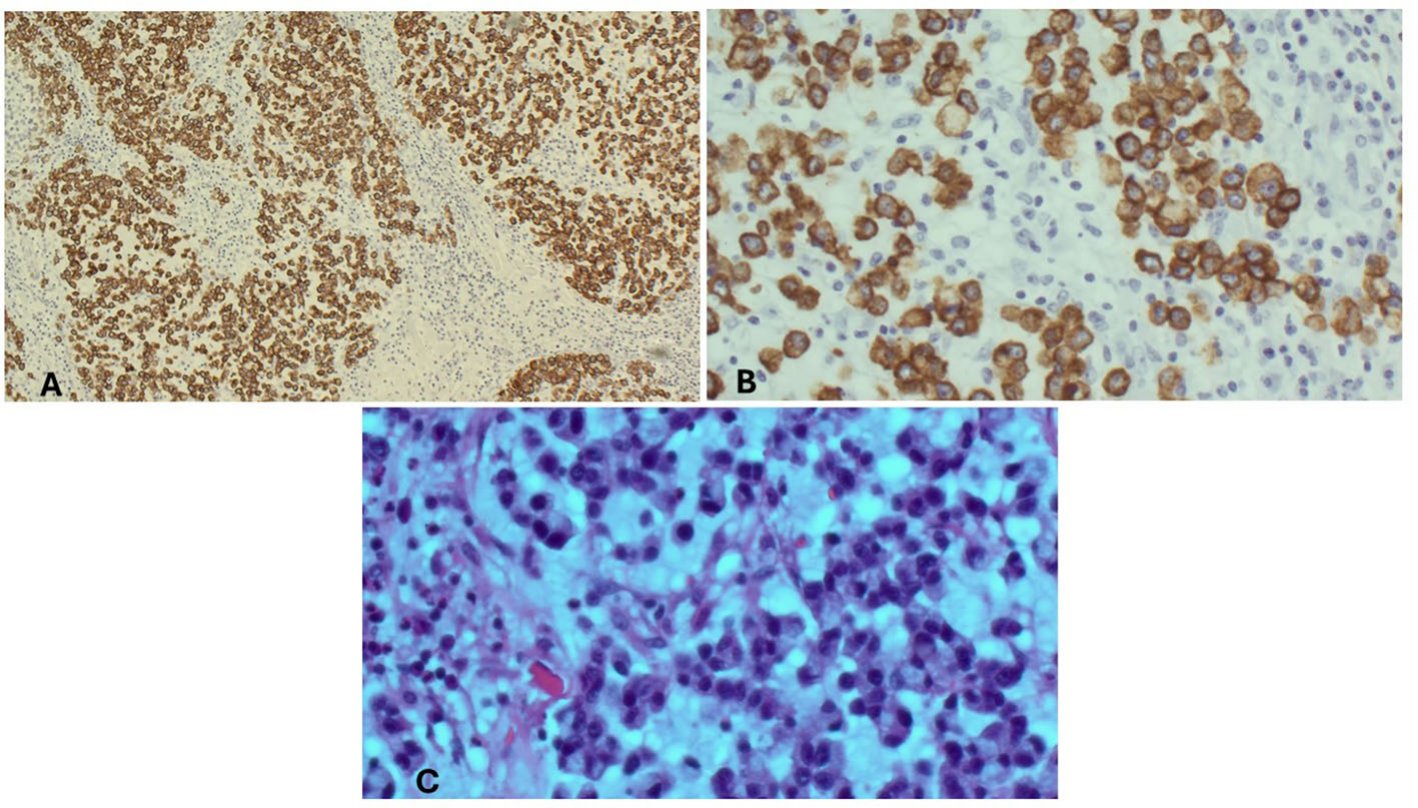
Microscopic findings of the abdominal wall mass. **(A)** Abdominal wall mass, immunohistochemical stain, CAM 5.2/AE1. **(B)** Abdominal wall mass, high power. **(C)** Abdominal wall mass, high power, H&E.

A healthy 59-year-old woman with a history of open cholecystectomy in a different country in September of 2022 first noticed a small painless mass in the right lower quadrant of her abdomen 5 months after her cholecystectomy. She presented to a clinic in that same country 10 months after her cholecystectomy for this abdominal wall mass, where she subsequently underwent biopsy. Shortly after this biopsy was completed, the patient presented to a US hospital for treatment of an infection at the biopsy site, where a repeat biopsy was obtained, which showed signet ring cell adenocarcinoma likely of gastrointestinal primary source. CT of the abdomen and pelvis obtained at that time showed a lobulated 5-cm lesion within the right mid-abdominal ventral wall with surrounding fat stranding and overlying soft tissue thickening (**Figure 1**), as well as an additional nearby structure noted as a possible lymph node or urachal cyst discovered near the urinary bladder. The patient underwent subsequent workup, including esophagogastroduodenoscopy (EGD), colonoscopy, and CT of the chest, abdomen, and pelvis, to identify a possible primary source, with no appreciation of a primary lesion. After multidisciplinary tumor board discussion and recommendation, the patient also underwent abdominal and pelvic ultrasound with no discovery of a primary mass. She then underwent diagnostic laparoscopy 12 months after her initial gallbladder resection, which was negative for any appreciable lesions or peritoneal carcinomatosis; however, a pedunculated uterine lesion was discovered and removed en bloc. At this time, the abdominal wall mass, which was noted to lie 3 cm inferior to the patient’s open cholecystectomy incision scar, was removed en bloc, including a portion of the anterior abdominal fascia, and was measured to be 8 × 6 × 3.5 cm. The final pathology of the uterine lesion noted a benign leiomyoma. The abdominal wall mass pathology described adenocarcinoma with signet ring and mucinous features with an undetermined primary site but favored upper gastrointestinal (GI) or pancreatic-biliary etiology as the most likely primary source (**Figure 2**).

**Figure 3:**
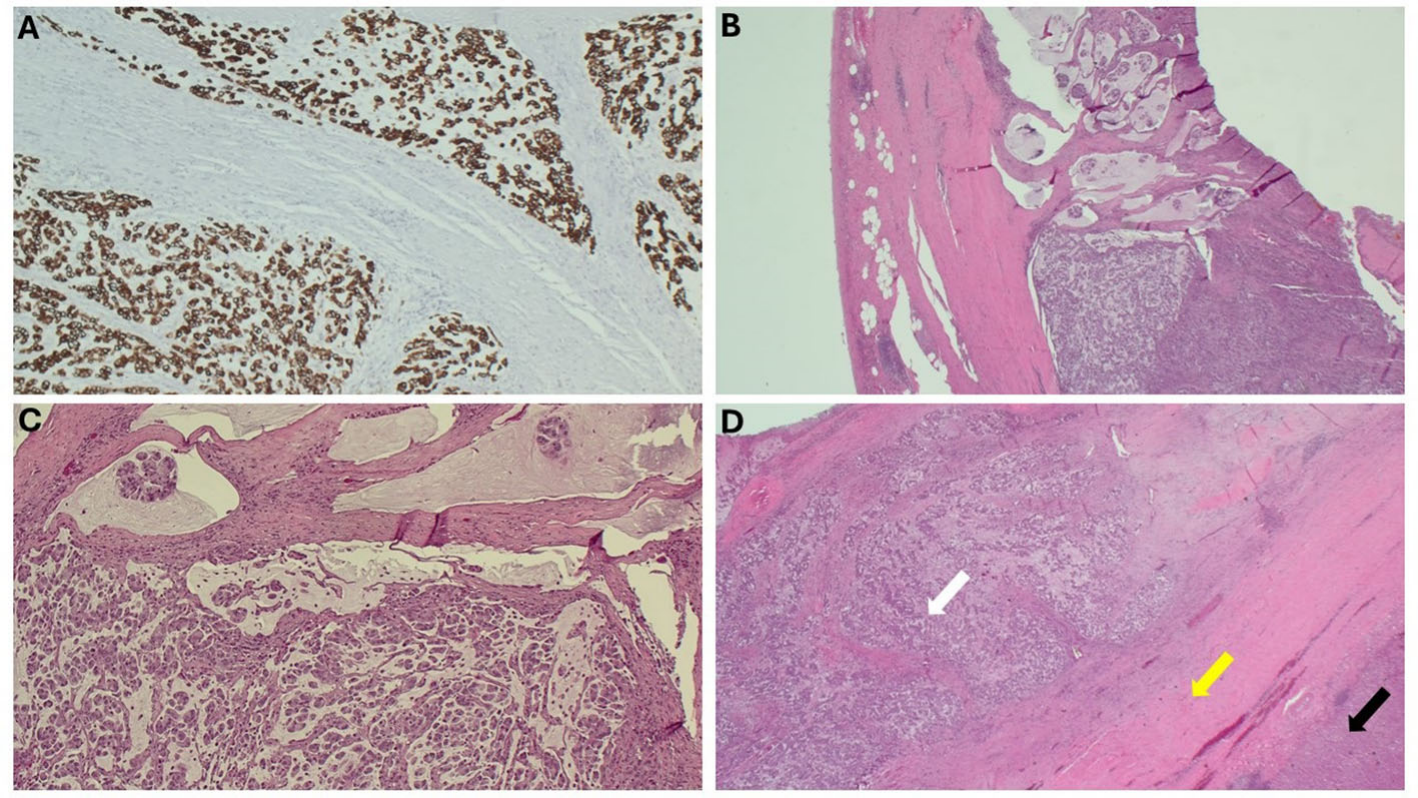
Microscopic findings of the gallbladder. **(A)** Gallbladder, immunohistochemical stain, CAM 5.2/AE1. **(B)** Gallbladder wall, H&E. **(C)** Gallbladder with mucinous pools of tumor, H&E. **(D)** Gallbladder wall (yellow arrow) with tumor infiltrating the wall (white arrow) and attached liver (black arrow), H&E.

Due to the proximity of the patient’s open cholecystectomy incision scar to the resected abdominal wall mass, further history was elucidated, and it was discovered that the patient had possession of her gallbladder in formalin. The specimen was obtained and sent for pathological evaluation 19 months after her initial gallbladder resection. The specimen was noted to have been previously sectioned, and pathology results reported moderately differentiated invasive mucinous adenocarcinoma involving nearly the entire muscular wall of the gallbladder, with attached hepatic parenchyma negative for invasive adenocarcinoma (**Figure 3**). The histology of the gallbladder tumor was noted to be essentially identical to that of the prior abdominal wall mass specimen. The patient was determined to have T1b disease based on this pathology; however, after further multidisciplinary tumor board discussion was held, she was considered to have metastasis to her abdomen due to her abdominal wall seeding and was thus classified as stage IVb disease. Because over 3 months had passed since her index gallbladder resection without evidence of other diseases, the decision was made to forgo adjuvant chemotherapy. Further management consisted of observation with physical exams and laboratory tests every 3 months. The most recent CT scan of the abdomen and pelvis, completed 11 months after resection of the abdominal wall lesion, showed no evidence of tumor recurrence or metastatic disease. The patient’s survival is 2 years 7 months to date after index cholecystectomy (**Table 1**).

**Table 1:** Clinical timeline.

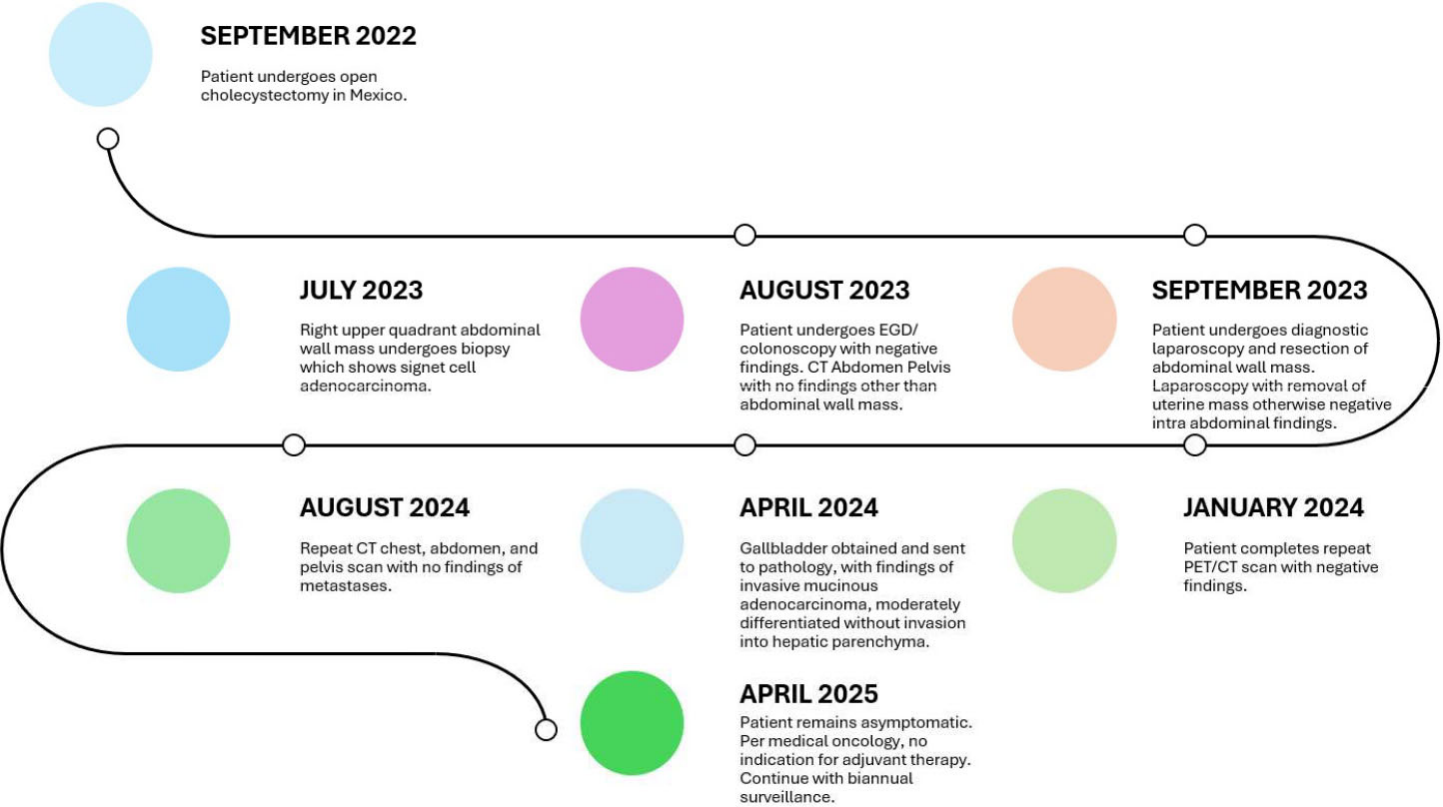

## Discussion

Port-site seeding most commonly occurs after resection of T2 and T3 gallbladder carcinoma (7) and is identified at a median of 180 days after laparoscopic gallbladder removal (4). Our case describes the symptomatic emergence of incisional site seeding 5 months after the index removal of pathological T1b gallbladder carcinoma. Our patient’s survival to date of 2 years 7 months after index cholecystectomy, as well as her lack of gross peritoneal disease, is unique compared to our literature review. In a systematic review by Berger-Richardson et al., 70% of the patients identified with port-site metastasis died at a median of 13 months after initial gallbladder resection, and 92% died within 2 years of resection. However, of the 8% (16 patients) who survived to 2 years after gallbladder resection, 14 of these patients had no evidence of disease recurrence after resection of the involved port sites. Berger-Richardson et al. used this information to advocate for resection of port-site metastasis in select cases where there is an absence of diffuse unresectable disease; however, they noted that their results indicate that the majority of patients who present with port-site metastasis are associated with diffuse peritoneal disease and early death (5). Other studies report no survival benefit in patients with port-site metastasis who undergo port-site excision (6, 7). Regardless, the presence of port-site seeding portends a worse overall prognosis (7) and thus warrants extensive workup for peritoneal disease and other tumor sites. In our case, this extensive workup was carried out prior to the knowledge of a primary gallbladder carcinoma diagnosis in attempts to locate the origin of the initial abdominal wall lesion of unknown origin.

Our case presents a unique management challenge because of the timing of retrospective gallbladder carcinoma diagnosis. Current surgical approaches for gallbladder carcinoma include extended cholecystectomy with segment IVb and V liver resection for T1b and T2a disease, and major hepatectomy, extended lymphadenectomy, and common bile duct resection with hepaticojejunostomy Roux-en-Y for T3 disease (9). Gallbladder carcinoma involving invasion to the hepatic artery, portal vein, or two or more adjacent organs is considered T4 disease and is generally deemed inoperable and managed instead with palliative systemic treatment (9). Time to oncologic resection from incidental discovery of carcinoma after laparoscopic cholecystectomy is usually 4 to 12 weeks (9), and because symptoms of port-site seeding generally do not present until approximately 25 weeks after initial gallbladder resection (4), the discovery and management of port-site seeding are usually approached after the initial oncologic surgical intervention has been completed. However, in the case that we present, the diagnosis of gallbladder cancer was made after the incision seeding site was discovered and resected. Because of this retrospective diagnostic timing, the abdominal wall lesion was considered to be a site of metastasis and thus classified as stage IV disease and was deemed not a candidate for liver resection.

Literature discussing presentation, management, and prognosis of patients with port-site seeding is largely based on studies involving laparoscopic gallbladder removal, while our case involves a patient who underwent open cholecystectomy. However, a systematic review carried out by Li et al. (2024) included 16 independent studies regarding the presence of port-site metastasis after resection of gallbladder cancer, and no significant difference in port-site metastasis was observed between patients undergoing laparoscopic surgery or open surgery, with an odds ratio of 1.597 (p > 0.01) (10). In regard to risk-reduction strategies, it has been shown that biliary tract violation is associated with increased risk of peritoneal carcinomatosis (11), although some literature also shows no difference in the survival curve for those with gallbladder cancer and intra-operative spillage during removal compared to those without spillage (12). While the use of retrieval bags in the removal of gallbladder cancer has been recommended in the past to prevent port-site seeding, recent studies found that approximately 50% of port-site seeding cases are appreciated at “non-extraction” port sites and that there are many reported cases of port-site metastasis in instances where retrieval bags were still used (4, 5). Because the original open cholecystectomy operative report was unable to be obtained for the patient in our case, specifics of the procedure are unknown including whether a laparoscopic approach was initially pursued, if a retrieval bag was used for gallbladder removal, if a wound protector was used, if bile spillage occurred, or if there were any gross signs intra-operatively suspicious for malignancy.

One limitation in the analysis of this case lies in the delayed pathological review of the specimen after initial resection, as some argue that tissue degradation in formalin may affect histologic accuracy. A retrospective study completed in 2022 by Likhithaswamy et al. evaluated tissues stored in formalin for at least 5 years compared to paraffin-embedded tissue blocks stored for 5 years, and it was found that while specimens can remain suitable for histopathological analysis after long-term storage in formalin, the specimens in formalin did demonstrate some changes in color, consistency, tissue integrity and architecture, and nuclear and cytoplasmic features. This study concluded that tissues are better preserved for long-term evaluation in paraffin blocks than in formalin for extended periods of time; however, if this is not an option, the specimens stored in formalin for long periods of time should undergo regular changes of solution with reliable pH maintenance (13). In our presented case, the specimen had been stored in unchanged formalin for 19 months after initial tissue resection, and the temperature or environment of the formalin-preserved specimen was unknown. Additionally, the initial cholecystectomy and abdominal wall biopsy performed abroad pose an ethical dilemma in the review of this case, given that the results of these pathologies were not disclosed to the patient. When evaluated by our institution’s pathology team, the gallbladder was noted to have been previously sectioned, insinuating that it was likely evaluated pathologically at the facility abroad. If the gallbladder underwent pathological evaluation and malignancy was determined, the question arises why the patient was not notified of these results. While this patient was notified of her diagnosis immediately at our institution, and institutional review board (IRB) approval and consent were obtained for the publication of this case, it is questionable whether the evaluation of the initial gallbladder specimen and abdominal wall biopsy completed abroad adhered to the same ethical standards as those within our institution.

Despite advances in research, diagnostic modalities, and interventions continuing to progress in the field of oncology, clinicians will continue to face complex oncologic scenarios in which treatment guidelines do not yet exist. Multidisciplinary tumor boards have long been present to address such issues, and teams comprised of a wide variety of clinicians including medical oncologists, radiation oncologists, surgical oncologists, radiologists, pathologists, pharmacists, and nutritionists are now more important than ever in creating a treatment approach for complex oncologic presentations lacking established guidelines (14, 15). Our rare case highlights the importance of a multidisciplinary tumor board approach to unprecedented oncology presentations, and we hope that this case will assist in the approach and decision-making process in future rare presentations.

## Conclusion

The presence of port-site metastasis after cholecystectomy is an important factor in the overall treatment and prognosis of gallbladder carcinoma; however, recommendations for the definitive management of these metastasis sites do not yet exist and remain controversial. The management of our unprecedented case of gallbladder carcinoma diagnosed retrospectively after the initial discovery and excision of incisional site metastasis is a difficult one, and the management of unique oncologic scenarios should utilize a multidisciplinary tumor board approach.

## Data Availability

The original contributions presented in the study are included in the article/supplementary material. Further inquiries can be directed to the corresponding author.
